# Another Brick to Confirm the Efficacy of Rigosertib as Anticancer Agent

**DOI:** 10.3390/ijms24021721

**Published:** 2023-01-15

**Authors:** Alessio Malacrida, Marie Deschamps-Wright, Roberta Rigolio, Guido Cavaletti, Mariarosaria Miloso

**Affiliations:** Experimental Neurology Unit, School of Medicine and Surgery, University of Milano-Bicocca, Via Cadore 48, 20900 Monza, MB, Italy

**Keywords:** rigosertib, lung cancer, glioblastoma, p53, cell cycle

## Abstract

Rigosertib is a small molecule in preclinical development that, due to its characteristics as a dual PLK1 and PI3K inhibitor, is particularly effective in counteracting the advance of different types of tumors. In this work, we evaluated the efficacy of Rigosertib and the expression of p53 in five different human tumor cell lines in vitro, A549 (lung adenocarcinoma), MCF-7 and MDA-MB231 (breast cancer cells), RPMI 8226 (multiple myeloma), and U87-MG (glioblastoma). We demonstrated that in all cell lines, the effect was dose- and time-dependent, but A549 cells were the most sensible to the treatment while higher concentrations were required for the most resistant cell line U87-MG. Moreover, the highest and lowest p53 levels have been observed, respectively, in A459 and U87-MG cells. The alterations in the cell cycle and in cell-cycle-related proteins were observed in A549 at lower concentrations than U87-MG. In conclusion, with this article we have demonstrated that Rigosertib has different efficacy depending on the cell line considered and that it could be a potential antineoplastic agent against lung cancer in humans.

## 1. Introduction

Rigosertib (ON-01910) (Rig) is a promising small molecule involved in several clinical trials as an anti-cancer drug [1]. It was initially described as a multi-kinase inhibitor with effects mainly on the PLK1 and PI3K pathways [1,2,3]. However, the real mechanism that explain the effectiveness of Rig in vitro and in vivo is not yet fully understood [4]. Several studies have highlighted that the RAS pathway appears to be one of the most affected by Rig [5,6,7]. The inhibition of the RAS/MEK/ERK pathway could be direct, with Rig acting as a RAS-mimetic [8], or indirect and dependent on oxidative stress [9]. Rig has been also proposed as a microtubule-destabilizing agent [10,11].

Moreover, Rig is capable of inducing senescence or cell death by apoptosis in several tumor cell lines, both in vitro and in vivo [4,6,12,13,14,15,16,17,18], increasing the production of ROS and altering the expression of numerous proteins involved in apoptosis (p53, BAX, MDM2) and in the formation of metastases (E-cadherins, matrix metalloproteinases 2 and 9) [5].

In our previous works, we have demonstrated that Rig is able to impair cell viability of human colangiocarcinoma EGI-1 cells in vitro [18]. Furthermore, due to its ability to block cells in the G2/M phase of cell cycle, Rig resulted also in a potent radiosensitizing agent [12,18]. Moreover, we also demonstrated that the main pathway of the Rig mechanism against EGI-1 cells included proteins involved in the regulation of the cell cycle and p53 modulation [19].

In the last few years, Rig has been evaluated in several clinical trials for the treatment of myelodysplastic syndromes, alone or in combination with other drugs [20,21,22,23]. Furthermore, various articles in literature have demonstrated the efficacy of Rig in vitro also in different types of solid tumors, such as colorectal cancer (SW48 and CaCo2 cells), neuroblastoma (LU-NB1, LU-NB2, LU-NB3 cells), retinoblastoma (Y79 and patient derived cells), hepatocellular carcinoma (Hep3B cells), head and neck cancer (UM-SCC cells), and colangiocarcinoma (EGI-1 cells) [6,14,15,16,17,18]. The aim of our work is to investigate if Rig could represent an effective anticancer agent also in other type of tumor lines. For this reason, in the present study, we investigated the efficacy of Rig against five different cell lines in vitro: human lung adenocarcinoma A549 (non-small-cell lung cancer), human breast cancer MCF-7 and MDA-MB231, human multiple myeloma RPMI 8226, and human glioblastoma U87-MG. We have chosen these lines for their differences in origin, morphology, and types of therapies available. Among them, we then focused our study on the A549 and U87-MG cell lines which represent the tumors with a growing incidence of drug resistance and high diffuse infiltrative nature. As current standard therapeutic options, they have limited effectiveness and it is therefore necessary to find more treatment strategies and drugs for improved survival and quality of patient’ life.

## 2. Results and Discussion

### 2.1. Effects of Rigosertib on Cell Viability of Different Tumoral Cell Lines

**Figure 1:**
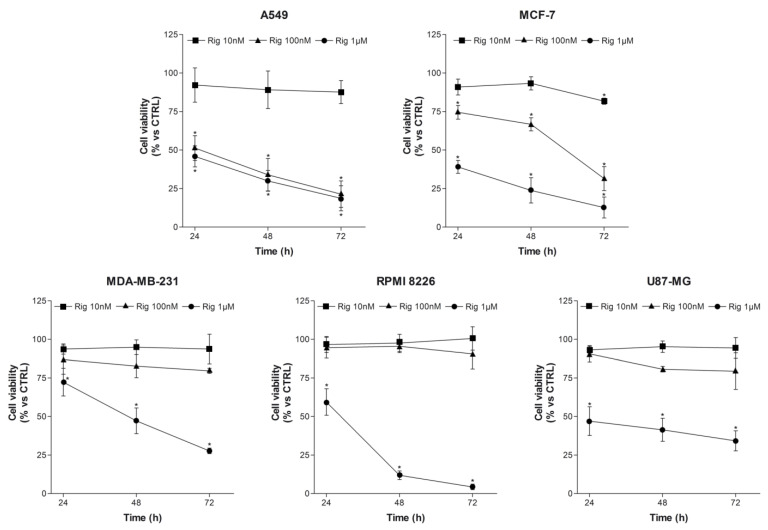
Evaluation of cancer cell viability and death after Rig treatment. Cell viability of A549, MCF-7, MDA-MB-231, RPMI 8226, and U87-MG cells was evaluated by MTT assay after 24, 48, and 72 h of treatment, with or without different concentrations of Rig (Rig 10 nM, 100 nM, 1 µM). * *p* < 0.05 vs. CTRL.

A549 (human lung adenocarcinoma), MCF-7 and MDA-MB-231 (humand breast cancer), RPMI 8226 (human multiple myeloma), and U87-MG (human glioblastoma) were used to evaluate the efficacy of Rig against different types of tumors. Cells were treated with different concentrations of Rig (10 nM, 100 nM, and 1 µM) for 24, 48, and 72 h. Cell viability was evaluated by MTT assay (Figure 1). Evaluated tumor cells responded differently to the treatment, with varying degrees of sensitivity to the Rig. A459 was the most sensible cell line to Rig treatment. The reduction of A549 cell viability was dose- and time-dependent, with an IC_50_ approximatively less than 100 nM for all the considered time points (Figure 1). Both breast cancer cells, MCF-7 and MDA-MB-231, also responded to Rig treatment in a dose- and time-dependent manner, but MCF-7 was more sensible to Rig treatment. RPMI 8226 and U87-MG were the most resistant cells to Rig. In fact, their cell viability was significantly reduced only at the highest concentration of Rig evaluated (1 µM) (Figure 1). However, RPMI 8226 cell viability was more reduced by Rig 1 µM than U87-MG.

### 2.2. Evaluation of p53 Expression in Different Tumoral Cell Lines and Its Role in Rigosertib Effect

**Figure 2:**
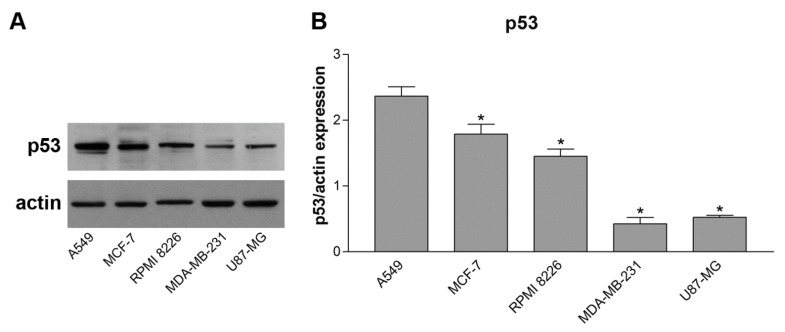
p53 protein expression in different cell lines after 24 h in culture medium. p53 protein expression of A549, MCF-7, RPMI 8226, MDA-MB-231, and U87-MG cells was quantified after western blot. (**A**) Representative images of p53 western blot. (**B**) Graph represents the quantification of p53 expression, normalized to actin, and data are expressed as the mean ± SD of three independent experiments. * *p* < 0.05 vs. A549.

As previously demonstrated, p53 was expressed in the human cholangiocarcinoma EGI-1 cell line and plays a key role in the Rig mechanism of action [19]. We evaluated p53 expression in A549, MCF-7, MDA-MB-23, RPMI 8226, and U87-MG cells. Cells were cultured in medium without any type of treatment for 24 h and western blot experiments were performed. As shown in Figure 2, between all the cell lines evaluated, A549 cells had the highest expression of p53 (comparable to the levels of EGI-1 cells in our previous paper [19], data not shown). Instead, MDA-MB-231 and U87-MG cells presented the lowest level of expression of p53.

**Figure 3:**
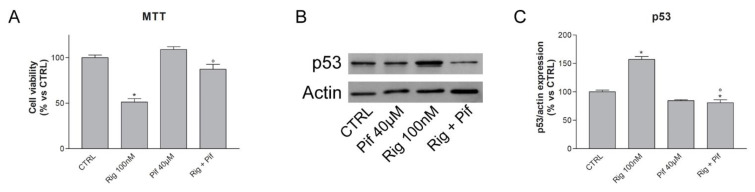
Evaluation of cancer cell viability and p53 expression after Rig and Pif treatment. (**A**) Cell viability of A549 cells was evaluated by MTT assay after 24 h of treatment, without treatments (CTRL), with Rig 100 nM, Pif 40 µM, or combination of Rig and Pif. (**B**) Representative images and (**C**) respective graph of p53 western blot of A549 cells treated for 24 h, without treatments (CTRL), with Rig 100 nM, Pif 40 µM, or combination of Rig and Pif. Graph represents the quantification of p53 expression, normalized to actin. * *p* < 0.05 vs. CTRL, ° *p* < 0.05 vs Rig 100 nM.

To demonstrate the role of p53 in the Rig effect, A549 cells were treated with the combination of Rig and Pifithrin α (Pif), an inhibitor of p53, which inhibits the transactivation of p53-responsive genes. As shown in Figure 3A, Pif almost completely reversed the effect of Rig 100 nM on A549 cell viability at 24 h of treatment. Moreover, as demonstrated by western blotting experiments, Rig 100 nM induced a significant increase in p53 protein expression after 24 h of treatment. Adding Pif to Rig treatment inhibited the increase of p53, and the levels of the protein were almost comparable to untreated controls (Figure 3B,C).

As early as 2014, Xu and collaborators hypothesized that the activation of p53 and its pathway were involved in the Rig effect against myelodysplastic syndrome [24].

Since the expression levels of p53 at the physiological level seem to correlate with the sensibility of cells to the treatment with Rig, we suggest that this protein is fundamental in the mechanism of action of the molecule. It will be important in the future to evaluate whether p53 mutations can somehow affect the efficacy of Rig against cancer cells.

### 2.3. Evaluation of Rigosertib-Induced Cell Death in A549 and U87-MG Cells

Lung cancer is one of the main cause of cancer-related deaths and represents one of the most frequent neoplasms in the world [25]. This type of tumor can be classified in two main types according to the histological features and pathological manifestations: small-cell lung cancer (SCLC) and non-small-cell lung cancer (NSCLC), the most frequent one, accounting for about 85% of cases [26]. To date, the main methods of treatment of NSCLS are surgical resection, radiotherapy, chemotherapy (Cisplatin and Carboplatin), or combinations of the above-mentioned methods [27]. Moreover, targeted therapy could be an option for treating lung NSCLS. For example, tyrosine kinase inhibitors (Gefitinib and Erlotinib) or ALK inhibitors (Crizotinib and Alectinib) can be used to treat patients with EGFR (10–15%) and ALK (3–7%) gene mutations, respectively [28,29,30,31]; monoclonal antibodies against PD-1 (Nivolumab) and PD-L1 (Atezolizumab) are used as immune checkpoints inhibitors and they have greatly improved the chance of treatment success of NSCLC in recent years [32,33,34].

However, these treatments frequently present debilitating side effects (diarrhea, pulmonary inflammation, polyneuropathies, cytopenia, and nephrotoxicity) which require a dose reduction or total interruption of the treatment [27,32]. Furthermore, tyrosine kinase and ALK inhibitors may not be effective in all patients. In some cases, however, these therapies are not sufficient due to drug resistance, low survival rate, and formation of metastases [35].

Glioblastoma is the most frequent brain neoplasm. It has a very rapid course and, due to late diagnosis and lack of effective therapies, its mortality rate is very high [31,32]. The first-line treatment in this type of tumor is surgical resection, although it can often be followed by radiotherapy and chemotherapy. The latter currently offers few effective options, among which temozolomide and nitrosoureas are the most used. This lack of chemotherapy options is mainly due to the high heterogeneity of the tumor, the abundance of cancer stem cells, the high drug resistance, and the difficulty of crossing the blood–brain barrier for many anticancer drugs [32].

In previous paragraphs, we demonstrated that: (1) A549 were the most sensible cells to the treatment with Rig, while U87-MG were the most resistant; and (2) A549 cells had the highest expression of p53, while U87 had the lowest expression. We therefore decided to focus our study on these two tumor cell lines evaluating the differences observed following treatment with Rig.

Trypan blue vital count was performed to analyze cell viability and death after Rig 100 nM treatment (which approximatively corresponds to the IC_50_ of Rig against A549 cells at 24 h).

**Figure 4:**
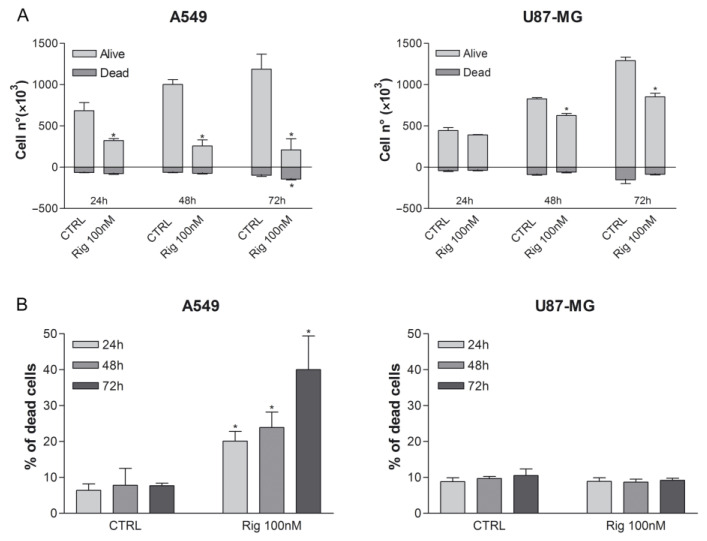
Evaluation of cancer cell viability and death after Rig treatment. (**A**) The number of viable and dead A549 and U87-MG cells, not treated (CTRL) or treated with Rig 100 nM for 24, 48, and 72 h, was evaluated by Trypan blue vital count assay. All the results are expressed as the mean ± SD of at least three independent experiments. * *p* < 0.05 vs. CTRL. (**B**) Percentage of dead A549 and U87-MG cells, not treated (CTRL) or treated with Rig 100 nM for 24, 48, and 72 h, was calculated on the results of Figure 3B. The percentage was calculated on the total of counted cells (arbitrarily set to 100%). * *p* < 0.05 vs. CTRL.

As observed in MTT assay, Rig 100 nM induced a time-dependent reduction of viable A549 cells. Contrariwise, in U87-MG, the reduction of cell viability was lower and significant only at 48 and 72 h (Figure 4A). 

Moreover, we calculated the percentage of dead cells compared to the sum of all counted cells. Untreated cells had a physiologic percentage of dead between 5 and 9% (depending on cell line). This percentage significantly increased in a time-dependent manner for A549 cells, reaching a maximum of 40% after 72 h of treatment. On the contrary, U87-MG dead cells were almost comparable to untreated controls, indicating a more cytostatic effect than cytotoxic (Figure 4B).

The 100 nm Rig concentration is consistent with Rig concentrations used in other studies; in fact, in all these works, Rig exhibited the same efficacy in reducing the viability of the cell lines evaluated, with effective concentrations starting approximately at 50–100 nM [6,14,15,16,17,18]. 

### 2.4. Cell Cycle Arrest in the G2/M Phase Induced by Rigosertib in A549 and U87-MG Cells

Cell cycle analysis of A549 and U87-MG cells were performed with a flow cytometer and propidium iodide staining. Cells were treated with increasing concentrations of Rig (10 nM, 100 nM, and 1 µM) for 24, 48, and 72 h and then cells were analyzed. 

**Figure 5:**
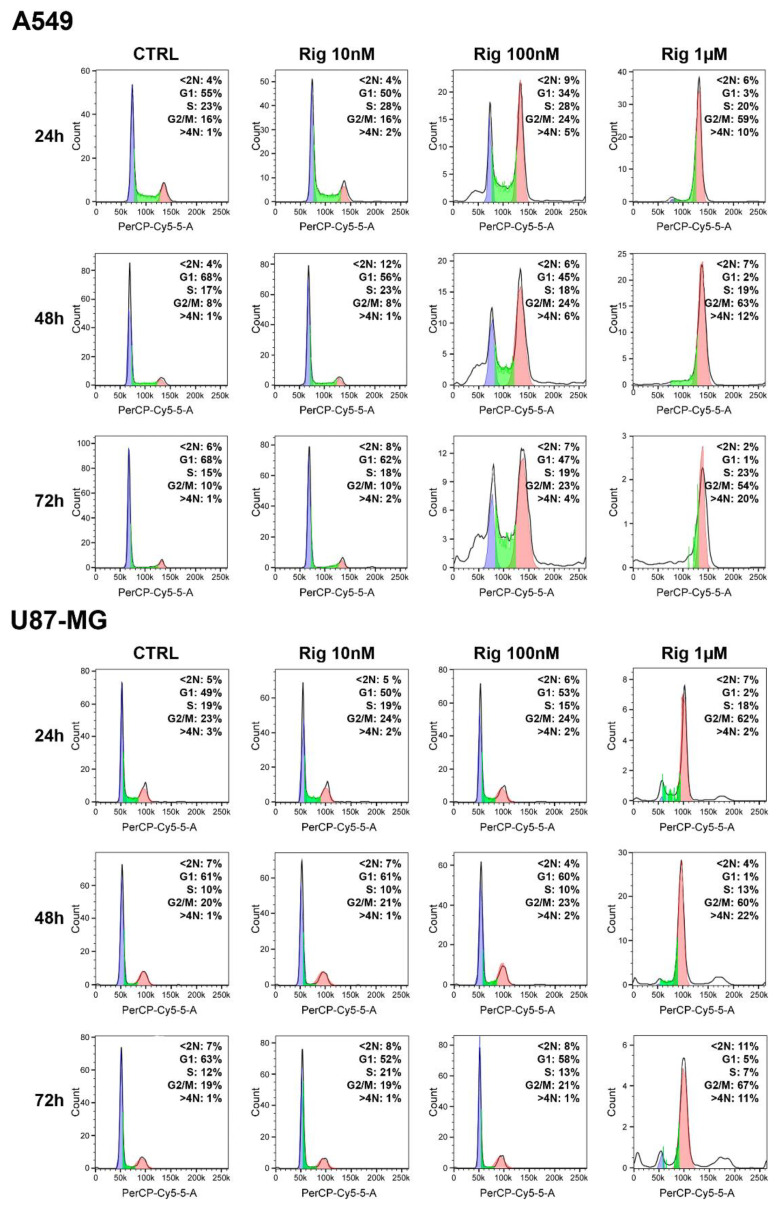
Cell cycle analysis of A549 and U87-MG cells after Rig treatment. Images are representative of cell cycle analysis of A549 and U87-MG, not treated (CTRL) or treated with increasing concentrations of Rig (10 nM, 100 nM, and 1 µM) for 24, 48, and 72 h. Blue, green and red colors represent cells in G1, S and G2/M respectively. The percentage of cell distribution in the different phases of cell cycle represented the average of at least three independent experiments.

In untreated A549, for all time points evaluated (24, 48, and 72 h), about 55–68% of cells were in the G1 phase, 15–23% in the S phase, and 8–16% in the G2/M phase, while less than 1% cells were polyploid. Compared to untreated cells, A549 cells treated with Rig 10 nM had a comparable distribution in the different phases of cell cycle for all the evaluated time points. Treatment of A549 cells with 100 nM Rig induced a decrease of cells in the G1 phase (34–47%), a slight increase in the S phase (18–28%), and an increase in the G2/M phase (23–24%). Increasing Rig concentration to 1 µM, the effect on the G1 and G2/M phases was very relevant and significant: cells in the G1 phase were less than 1–3%, while cells in the G2/M phase were about 54–63%. Cells in the S phase were slightly higher than controls (19–23%), while polyploid cells increased to 10–20% (Figure 5).

In untreated U87-MG cells and in cells treated with Rig 10 and 100 nM, cell cycle distributions were comparable for all the evaluated time points (24, 48, and 72 h): about 49–63% of cells were in the G1 phase, 10–21% in the S phase, and 19–24% in the G2/M phase, while only 1–3% of cells were polyploid. On the contrary, the effect of Rig 1 µM on cell cycle distribution was very significant. In fact, only 2–5% of cells were in the G1 phase and about 60–67% of cells were in the G2/M phase. Cells in the S phase were only slightly reduced compared to controls (7–18%), while polyploid cells increased to 11–22% at 48 and 72 h of treatment (Figure 5).

These results could explain, at least partially, the difference in Rig efficacy in the two cell lines evaluated. In fact, in A549 cells, important and significant alterations in the distribution in the cell cycle can already be observed at the concentration of 100 nM. A549 cells are more blocked in the G2/M phase and polyploid cells increased. On the contrary, higher concentrations are required for U87 cells to achieve the same cell cycle alterations. 

As widely reported in the literature, Rig is a molecule capable of inducing cell cycle arrest in the G2/M phase [12,18,36,37].

### 2.5. Modulation of the Expression of EMI1, Cyclin B, PLK1, and P-CDK1 Proteins Induced by Rigosertib in A549 and U87-MG Cells

In our previous work, we demonstrated that, in human EGI-1 human cholangiocarcinoma cells, Rig induced the modulation of EMI1, Cyclin B, PLK1, and P-CDK1 proteins [19]. The level of the same proteins was evaluated in A549 and U87-MG cells after treatment with increasing concentrations of Rig (10 nM, 100 nM, and 1 µM) for 24 and 48 h. 

**Figure 6:**
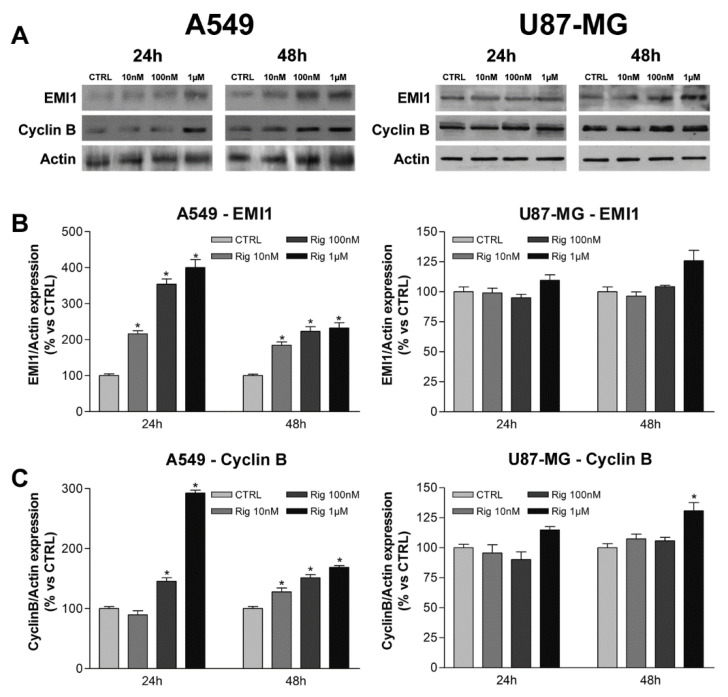
EMI1 and Cyclin B protein expression in A549 and U87-MG cells. (**A**) Representative images of EMI-1 and Cyclin B western blots of A549 and U87-MG cells. (**B**) EMI1 and (**C**) Cyclin B protein expression in A549 and U87-MG cells treated with increasing concentrations of RIG (10 nM, 100 nM, and 1 µM) for 24 and 48 h. Graphs represent the mean ± SD of protein expression normalized to actin and to untreated controls, set at 100%. (* *p* < 0.05 vs. CTRL).

In A549 cells, Rig increased EMI-1 and Cyclin B in a dose-dependent manner, but the increase observed at 24 h was more significant than at 48 h. In U87-MG cells, alterations in EMI-1 and Cyclin B protein expression induced by Rig were not significant at any concentration and time considered, except for Cyclin B at 48 h and 1 µM (Figure 6).

**Figure 7:**
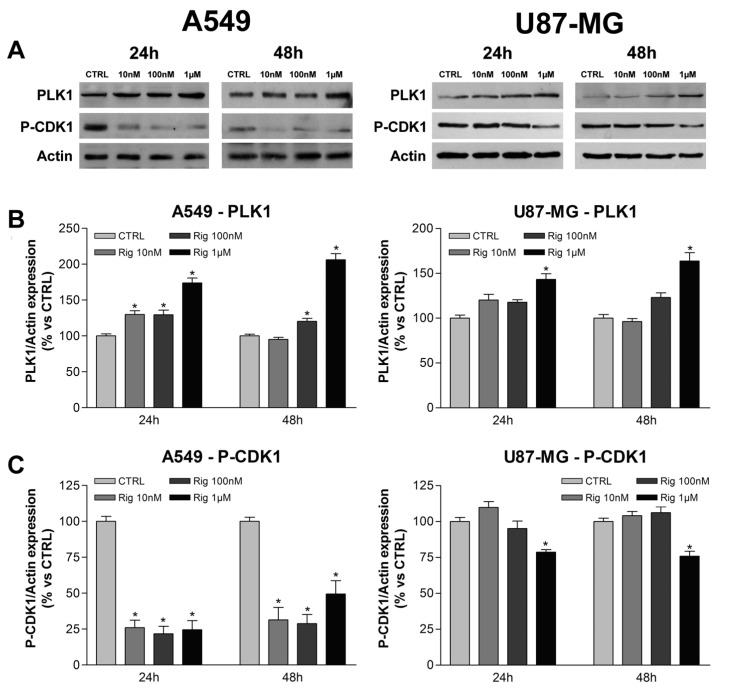
PLK1 and P-CDK1 protein expression in A549 and U87-MG cells. (**A**) Representative images of PLK1 and P-CDK1 western blots of A549 and U87-MG cells. (**B**) PLK1 and (**C**) P-CDK1 protein expression in A549 and U87-MG cells treated with increasing concentrations of RIG (10 nM, 100 nM, and 1 µM) for 24 and 48 h. Graphs represent the mean ± SD of protein expression normalized to actin and to untreated controls, set at 100%. (* *p* < 0.05 vs. CTRL).

In A549 cells, Rig induced a significant dose-dependent increase of PLK1 at both considered time points. In U87-MG cells a slightly increase in PLK1 protein expression was observed, becoming significant only at 1 µM concentration (Figure 7).

The effect of Rig on P-CDK1 in A459 and U87-MG is very different. In A549 cells, the decrease of CDK1 phosphorylation was significantly observed in all the times and Rig concentrations used. For U87-MG cells instead, no CDK1 phosphorylation reduction was observed for Rig 10 and 100 nM, while it was evident and significant only at 1 µM concentration (Figure 7). CDK1 total protein expression was not altered in any way by the treatment with Rig, at any time point evaluated and for both cell lines (data not shown).

What we observed here in A549 cells is quite comparable to what we demonstrated previously with EGI-1 cells [19]. These protein alterations could explain the fact that A549 and EGI-1 cells respond very similarly to the treatment with Rig. Conversely, U87-MG cells do not show significant alterations in the expression of the evaluated proteins, except at the 1 µM concentration. Even in this case, however, the increase (or reduction) observed is much lower than the ones of the other two cell lines. This could mean that the reduction of cell viability and the arrest in the G2/M phase of U87-MG cells at 1 µM Rig is due to another mechanism that will be important to evaluate in future works.

### 2.6. Cell Migration, but Not Cell Invasion, Is Impaired by Rigosertib in A549 and U87-MG Cells

Scratch wound healing and Boyden chamber assays were performed to evaluate the effect of Rig on cell migration and invasion. Human A549 and U87-MG cells were treated with increasing concentrations of Rig (10 nM, 100 nM, and 1 µM) for 24 h and then their ability of migration and invasion was evaluated. 

**Figure 8:**
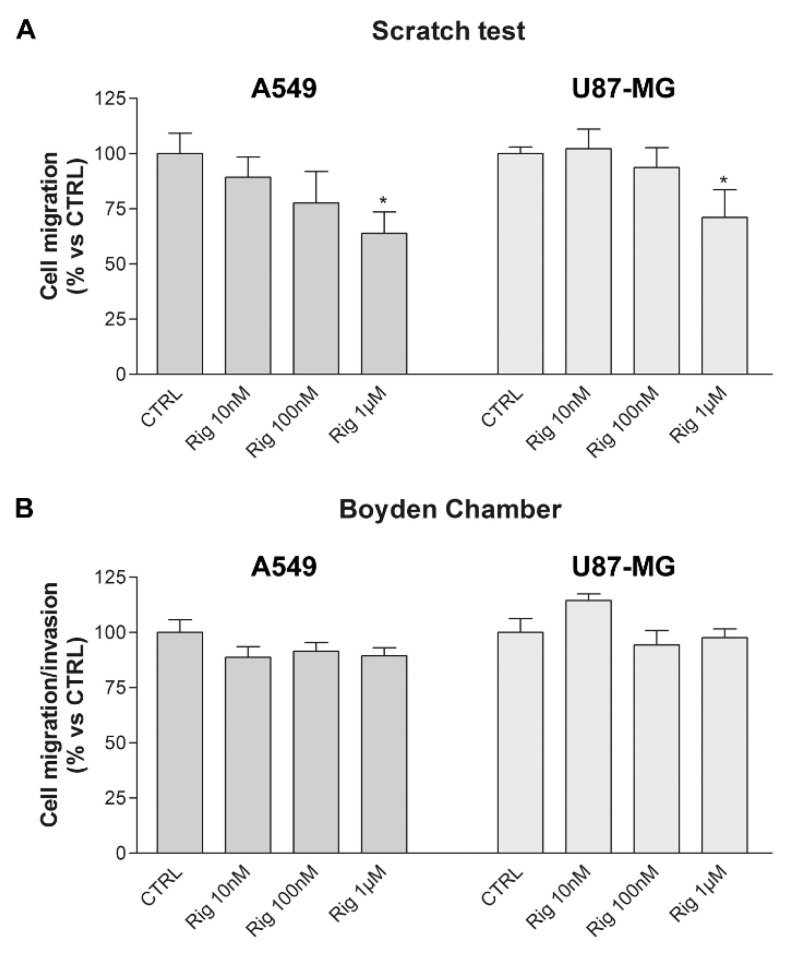
Migration and invasion of A549 and U87-MG cells after Rig treatment. (**A**) 24 h scratch wound healing assay of A549 and U87-MG cells after treatment with increasing concentrations of Rig (Rig 10 nM, 100 nM, and 1 µM). Graphs are represented as the mean percentage ± SD of at least three independent experiments and are compared to untreated controls (CTRL, arbitrarily set to 100%). * *p* < 0.05. (**B**) Boyden chamber assay of A549 and U87-MG cells after treatment with increasing concentrations of Rig (Rig 10 nM, 100 nM, and 1 µM) for 24 h. Data are normalized on cell viability. * *p* < 0.05.

In the scratch wound healing assay, Rig was able to slightly reduce the migration ability of A549 cells and U87-MG cells in a dose-dependent manner and the reduction was significant only after treatment with Rig 1 µM (Figure 8A).

In the Boyden chamber assay, both A549 and U87-MG cells, treated with all Rig concentrations and normalized on cell viability results, are not able to significantly reduce their invasiveness (Figure 8B). 

To date, there are not many data demonstrating the efficacy of Rig in reducing tumor cell migration in vitro. With the exception of a work by Chapman et al., in which Rig reduced leukemia cell migration by 80% at a 2 µM concentration [38], the only evaluation of this effect was conducted by us in our previous article [18]. These data could indicate that Rig is a molecule with a capacity to reduce the formation of metastases, but at concentrations higher than the ones able to achieve the reduction of cell viability.

## 3. Conclusions

In conclusion, in this work we have demonstrated that in vitro Rig is effective against human lung adenocarcinoma A549 cells. The modulation of different proteins evaluated suggests that the mechanisms involved seem to be comparable to those observed with EGI-1 cells. In the future, further investigations of Rig effects against lung cancer cells will be needed. It will be important to evaluate the effect of Rig on other lung carcinoma cell lines. Moreover, it will be very interesting to study how this promising molecule behaves in more complex conditions than simple cell cultures, such as 3D cultures, organoids, up to in vivo animal models. This last step will be essential for evaluating the potential use of this molecule as an antineoplastic agent in humans.

## 4. Materials and Methods

### 4.1. Cell Cultures and Reagents

Human lung adenocarcinoma A549 cells, human breast cancer MCF-7 cells, and human multiple myeloma RPMI 8226 cells (ATCC, Manassas, VA, USA) were cultured in RPMI 1640 medium supplemented with 10% fetal bovine serum (FBS), 1% L-glutamine, 1% penicillin, and streptomycin (Euroclone, Pero, Italy). Human breast cancer MDA-MB-231 and human glioblastoma U87-MG cells (ATCC, Manassas, VA, USA) were cultured in DMEM low glucose medium supplemented with 10% fetal bovine serum (FBS), 1% L-glutamine, 1% penicillin, and streptomycin (Euroclone, Pero, Italy). 

Rig, kindly provided by Onconova Therapeutics (Newtown, PA, USA), was resuspended in water at 105 mM concentration and then diluted to working concentrations directly in culture medium. 

### 4.2. MTT Assay

Cells were seeded at 10,000 cells/well density in 96-well plates and then treated with increasing concentrations of Rig. After 24, 48, and 72 h, a solution of MTT 0.5 mg/mL was added (Merck, Rahway, NJ, USA). After 4 h of incubation, formazan crystals were solubilized in EtOH and absorbance was measured using a microplate reader. 

### 4.3. Trypan Blue Vital Count Assay

Cells were seeded at 250,000 cells/well density in 6-well plates. After treatment, cells were incubated for 24, 48, and 72 h. Cells were then collected after trypsinization, resuspended in a Trypan blue solution, and counted in a hemocytometer. Both viable and dead cells were counted.

### 4.4. Scratch Wound Healing Assay

Scratch wound healing assay was performed to evaluate cell migration. Cells were seeded at high density in 6-well plate and incubated until confluence. Complete culture medium was replaced with serum-free medium. After 24 h, a scratch was made on the cell monolayer using a plastic tip; culture medium was replaced with fresh complete medium with or without treatments. Micrographs of the scratch were taken soon after the scratch and after 24 h. Area of migration of the cells were measured using ImageJ software (v1.53c). 

### 4.5. Boyden Chamber

Boyden chamber was performed to assess cell invasiveness. 10 × 103 cells were seeded in serum free-medium with or without treatments in the upper compartment of the Boyden chamber. In the lower compartment, a complete medium was placed and FBS was used as chemoattractant. Between the two compartments, a gelatin-coated polycarbonate membrane with 8 µm pores was placed. After 24 h of incubation, the membrane was removed, and the cells on the upper surface of the membrane were mechanically removed, while the cells on the lower surface were fixed and stained with diffQuick solutions. Stained cells were than counted.

### 4.6. FACS Cell Cycle Analysis

Cells were seeded at 250,000 cells/well density in 6-well plates and treated with increasing concentrations of Rig. After incubation, cells were collected after trypsinization, resuspended in a glucose saline solution, and fixed with ethanol for 24 h. Subsequently, cells were stained with a solution of propidium iodide and RNase and analyzed by flow cytometer (FACS Canto, BD Biosciences, San Jose, CA, USA).

### 4.7. Western Blotting

Cells were plated and treated as described in Trypan blue paragraph. After incubation with treatments, cells were chemically lysed with lysis buffer (Hepes pH7.5 1M, NaCl 3M, glycerol, Triton X-100 10%, MgCl_2_ 1.5M, EGTA 0.1M, PMSF 0.1M, aprotinine 1%, Na-pyrophosphate 0.1M, Na_3_VO_4_ 0.5M) and mechanically removed with a cell scraper. Protein samples were than clarified with centrifugation and quantified using Bradford method. Proteins were than separated in an SDS-PAGE and subsequently transferred to a nitrocellulose membrane. Western blotting was performed according to antibody manufacturers. Primary antibodies used: anti-EMI1 (1:1000, Santa Cruz Biotechnology, Dallas, TX, USA), anti-cyclin B (1:1000, Santa Cruz Biotechnology, Dallas, TX, USA), anti-PLK1 (1:1000, Santa Cruz, Dallas, TX, USA), anti-P-CDK1 (1:1000, Cell Signaling, Danvers, MA, USA), anti-p53 (1:1000), and anti-actin (1:2000). Secondary antibody: anti-rabbit (1:2000), anti-mouse (1:2000), and anti-goat (1:2000). To detect proteins, enhanced chemiluminescence was performed using the Lite Ablot PLUS kit (Euroclone, Pero, Italy) and images of western blot were analyzed with ImageJ software (v1.53c).

### 4.8. Data Representation and Statistical Analysis

Unless otherwise stated, graphs are represented as the mean percentage ± SD of at least three independent experiments and are compared to untreated controls (arbitrarily set to 100%). All data were analyzed using GraphPad Prism software (v3.0, Boston, MA, USA). Statistically significant differences between the control and the treatments were determined using a non-parametric Kruskal–Wallis test. Differences were considered statistically significant if *p* < 0.05. 

## Data Availability

Not applicable.
