# Correction: Malacrida et al. Another Brick to Confirm the Efficacy of Rigosertib as Anticancer Agent. *Int. J. Mol. Sci.* 2023, *24*, 1721

**DOI:** 10.3390/ijms27136071

**Published:** 2026-07-07

**Authors:** Alessio Malacrida, Marie Deschamps-Wright, Roberta Rigolio, Guido Cavaletti, Mariarosaria Miloso

**Affiliations:** Experimental Neurology Unit, School of Medicine and Surgery, University of Milano-Bicocca, Via Cadore 48, 20900 Monza, MB, Italy; mariedcpswright@gmail.com (M.D.-W.); roberta.rigolio@unimib.it (R.R.); guido.cavaletti@unimib.it (G.C.); mariarosaria.miloso@unimib.it (M.M.)

In the original publication [1], there was a mistake in the published version of Figure 7. An error occurred while generating Figure 7. Specifically, a mistake in the insertion and alignment of certain layers representing the Western blot bands of PLK1 at 48 h of both A549 and U87-MG resulted in an incorrect image being exported. The corrected Figure 7 appears below. The authors state that the scientific conclusions are unaffected. This correction was approved by the Academic Editor. The original publication has also been updated.

**Figure 7:**
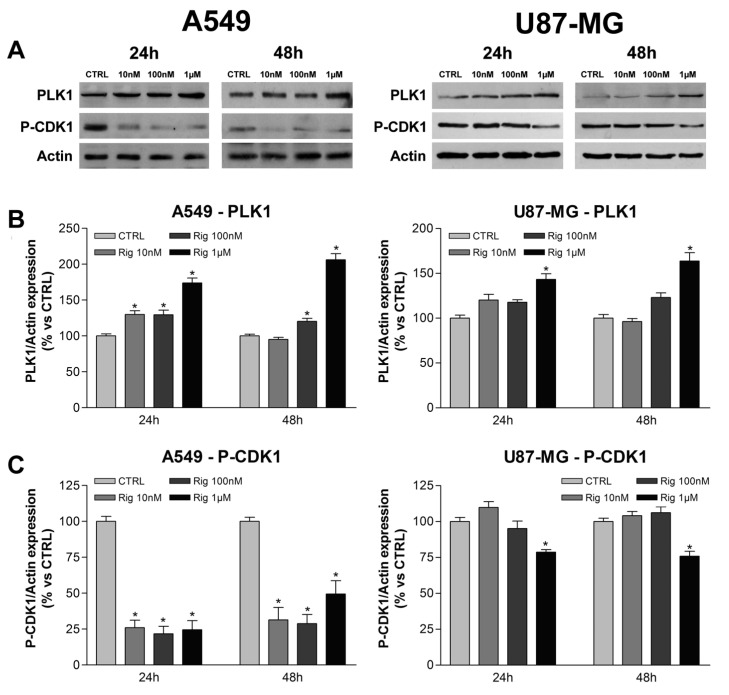
PLK1 and P-CDK1 protein expression in A549 and U87-MG cells. (**A**) Representative images of PLK1 and P-CDK1 western blots of A549 and U87-MG cells. (**B**) PLK1 and (**C**) P-CDK1 protein expression in A549 and U87-MG cells treated with increasing concentrations of RIG (10 nM, 100 nM, and 1 µM) for 24 and 48 h. Graphs represent the mean ± SD of protein expression normalized to actin and to untreated controls, set at 100%. (* *p* < 0.05 vs. CTRL).
